# Nasopupillary Asymmetry

**DOI:** 10.1155/2014/347826

**Published:** 2014-12-04

**Authors:** Eduardo Arenas, Diana Muñoz, Evelyn Matheus, Diana Morales

**Affiliations:** ^1^Department of Ophthalmology, Colombia National University, Bogota, Colombia; ^2^Department of Physiology, Colombia National University, Bogota, Colombia; ^3^Eduardo Arenas Ophthalmology Society, Bogota, Colombia

## Abstract

*Purpose*. To establish the prevalence of nasopupillary asymmetry (difference in nasopupillary distances) in the population and its relation with the interpupillary distance. *Methods*. A retrospective descriptive study was conducted by reviewing of 1262 medical records. The values of nasopupillary asymmetry and the interpupillary distance were obtained. A statistical analysis was made and the correlation between these variables was established. *Results*. Seventy-nine percent of the population presented some degree of nasopupillary asymmetry. The interpupillary distance had a very low correlation with the nasopupillary asymmetry (*r* = 0.074, *P* = 0.0). *Conclusion*. It is advisable to use the nasopupillary distance of each eye as a standard measurement.

## 1. Introduction

The interpupillary distance (ID) is the distance measured between the centers of the pupils, and it is important for the creation of the stereoscopic vision, which results in a single tridimensional image [1, 2]. The ID is an important measure widely used today as part of the process of eyeglasses formulation [3, 4]. Furthermore, the nasopupillary distance (NPD) is the measure from the pupil center to the medial nasal axis [5, 6].

However, currently the nasopupillary asymmetry (NPA) (difference in nasopupillary distances) and its relation to the ID have not been studied [7]. Additionally, there have not been reported in the literature data of the percentage of the population with NPA.

In this research, the values of NPA and its relation to ID in a Colombian population were studied, evidencing that most of the population presents some degree of NPA. This is the first research reported in the literature that studies the NPA.

## 2. Methods

A retrospective descriptive study was conducted by reviewing 1300 medical records. Patients with any type of strabismus, single eye and those under 18 were excluded, thereby obtaining a total sample of 1262 medical records of 631 women and an equal number of men, aged between 18 and 99 years.

**Figure 1:**
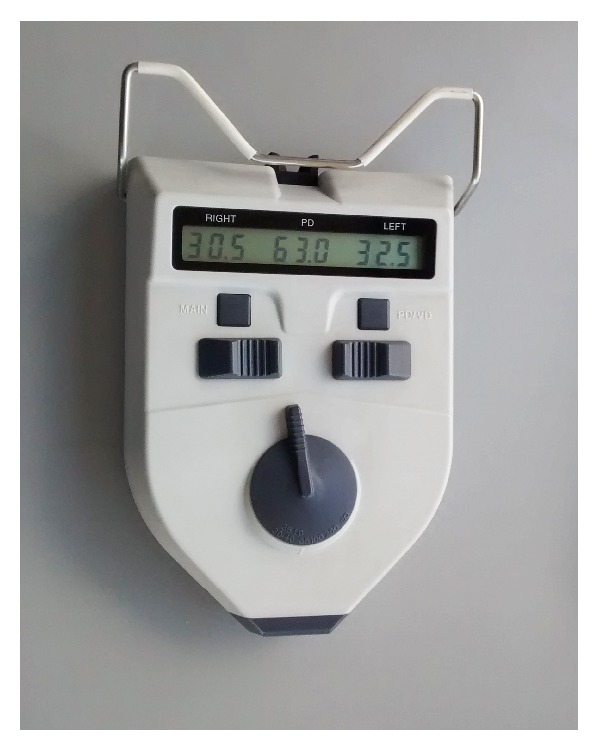
Digital pupillometer that shows in the center the ID and to the sides the NPD of each eye.

The ID and NPD measurement was performed all the time by the same specialist utilizing the Essilor pupillometer calibrated by long distance. The instrument shows the ID and also the two separate nasopupillary distances (right and left eyes) (Figure 1). The NPA was obtained by calculating the difference between the NPD of the right eye (RE) and the NPD of the left eye (LE), for each patient. Data was collected and a statistical analysis for the entire population was performed.

The degree of NPA was classified as low (up to 2 mm), medium (greater than 2 mm up to 4 mm), and high (greater than 4 mm). Also, which eye was farthest from the nasal axis (greater NPD) and the distribution of this variable to the entire population were analyzed. A calculation to determine the relation between the NPA and the ID was made, by Pearson's correlation coefficient.

## 3. Results

The average age of the population was 46.8 ± 18 (range: 18–99) years, with a mean of 48.65 ± 17.57 (range: 18–99) years for women and 44.94 ± 18.24 (range: 18–92) years for men. Of the total sample, 288 (22.82%) patients were from 18 to 30 years of age, 444 (35.18%) were from 31 to 50, 382 (30.26%) were from 51 to 70, and 148 (11.72%) were from 71 to 99.

The NPD mean of the RE was 31.65 ± 1.86 (range: 26–38) mm, median was 31.5 mm, and the mode was 31 mm. The NPD of the LE had a mean of 30.90 ± 1.8 (range: 26–37) mm, a median of 31 mm, and a mode of 31 mm.

For the ID a mean of 62.58 ± 3.35 (range: 52.5–73) mm, a median of 62 mm, and a mode of 62 mm were found. The mean of NPA for the population was 1.28 ± 1.04 (range: 0–6) mm. A median of 1 mm and a mode of 1 mm for the NPA in the population were obtained. The confidence interval for the NPA was 1.28 ± 0.057 mm.

Of all patients, 997 (79%) had some degree of NPA and 265 (21%) had a symmetrical NPD, and this difference was statistically significant (*P* = 0.0). 834 patients (83.73%) showed a low NPA, 155 (15.46%) a medium NPA, and 8 (0.8%) a high NPA.

**Figure 2:**
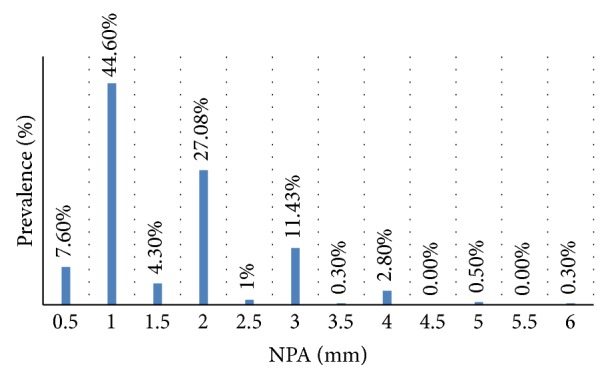
Distribution of the NPA in the population.

**Figure 3:**
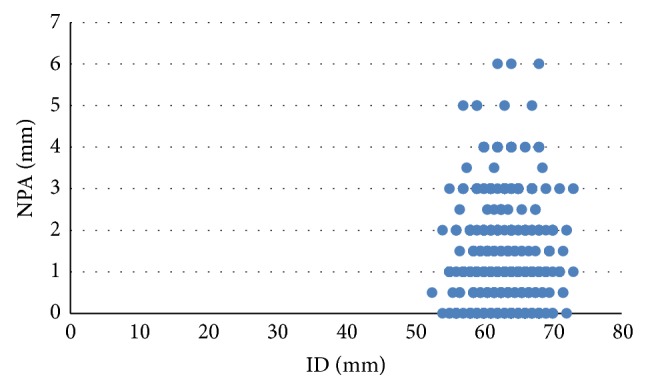
Correlation between the ID and the NPA. A very low correlation between both variables is observed (*r* = 0.074, *P* = 0.0).

Of the total population of patients with NPA, 76 (7.6%) patients had an NPA of 0.5 mm, 445 (44.6%) of 1 mm, 43 (4.3%) of 1.5 mm, 270 (27.08%) of 2 mm, 10 (1%) of 2.5 mm, 114 (11.43%) of 3 mm, 3 (0.3%) of 3.5 mm, 28 (2.8%) of 4 mm, 5 (0.5%) of 5 mm, and 3 (0.3%) of 6 mm (Figure 2). 729 patients (73.19%) had the RE farther than the LE (RE NPD greater than LE NPD) and the remaining 268 (26.8%) had the LE farther than the RE (LE NPD higher than the RE NPD) (*P* = 0.0). A very low correlation between ID and NPA was found (*r* = 0.074, *P* = 0.0) (Figure 3).

## 4. Discussion

The magnitude of ID has a great importance; however, the perfect symmetry on the face is not present in all people [8, 9]. The ID does not consider the ocular asymmetry. The nature of the NPD has elements that make it variable, such as the gender, race, and age [3, 10–12]. This variability may be influenced by the techniques and instruments used to measure it, due to parallax errors, patient care, reading accuracy, and the fixation point, especially when the millimeter ruler is used [13].

The pupillometer is a tool that can be used to measure both the NPD and the ID, in which the measurement is based on the assessment of the corneal reflex in both eyes while the patient observes a fixation point. This method provides a more accurate measurement than a metric ruler, since when the patient observes the fixation point, the corneal reflection denotes the point known as visual axis or vision line, and without the errors of parallelism of the lanterns or other systems [14].

In this paper the NPA was studied through a digital pupillometer in a Colombian population, finding that 79% of the population has some degree of NPA. 83.73% of the population with NPA presented a low NPA, which could not be clinically significant; nonetheless, the remaining 16.7% presented a greater NPA, which could be significant.

The low correlation found between the ID and the NPA (*r* = 0.074) reflects the lack of relation between the ID and the ocular asymmetry. This could be especially important for the eyeglasses prescription, since if this is not taken into account, it could cause the failure to obtain a correct centering of the ophthalmic lenses, inducing a prismatic effect and a binocular vision, especially in progressive lenses [15, 16].

All of the above underlines the importance of the NPD of each eye as an optometric measure. It should be noted that most automated refractometers and phoropters do not have the possibility of including the NPD at the time of examination.

According to our results it would be advisable to use the NPD of each eye as standard measurement, especially in patients with medium and high NPA, when the refractive error is high and of course with the increasing use of multifocal lens for patients with presbyopia [17]. Similarly, the relation of the NPA with other variables should be established. This will be studied by the present authors in a further study.
